# *Mycobacterium tuberculosis *infection induces non-apoptotic cell death of human dendritic cells

**DOI:** 10.1186/1471-2180-11-237

**Published:** 2011-10-24

**Authors:** Ruth CM Ryan, Mary P O'Sullivan, Joseph Keane

**Affiliations:** 1 Department of Clinical Medicine, Institute of Molecular Medicine, Trinity College Dublin, Ireland; 2 St. James's Hospital, Dublin 8, Ireland

## Abstract

**Background:**

Dendritic cells (DCs) connect innate and adaptive immunity, and are necessary for an efficient CD4^+ ^and CD8^+ ^T cell response after infection with *Mycobacterium tuberculosis *(Mtb). We previously described the macrophage cell death response to Mtb infection. To investigate the effect of Mtb infection on human DC viability, we infected these phagocytes with different strains of Mtb and assessed viability, as well as DNA fragmentation and caspase activity. In parallel studies, we assessed the impact of infection on DC maturation, cytokine production and bacillary survival.

**Results:**

Infection of DCs with live Mtb (H37Ra or H37Rv) led to cell death. This cell death proceeded in a caspase-independent manner, and without nuclear fragmentation. In fact, substrate assays demonstrated that Mtb H37Ra-induced cell death progressed without the activation of the executioner caspases, 3/7. Although the death pathway was triggered after infection, the DCs successfully underwent maturation and produced a host-protective cytokine profile. Finally, dying infected DCs were permissive for Mtb H37Ra growth.

**Conclusions:**

Human DCs undergo cell death after infection with live Mtb, in a manner that does not involve executioner caspases, and results in no mycobactericidal effect. Nonetheless, the DC maturation and cytokine profile observed suggests that the infected cells can still contribute to TB immunity.

## Background

Tuberculosis is responsible for 1.7 million deaths annually, and *Mycobacterium tuberculosis *(Mtb) infects up to one third of the world's population [1,2]. Yet the human host response to Mtb infection in 90% of cases is an immune success story; where infection is followed, not by disease, but by lifelong latent infection [1].

The key role played by dendritic cells (DCs) in this successful host response has been well studied [3]. After inhalation, Mtb bacilli are phagocytosed by alveolar macrophages and DCs resident in the alveolar space. It falls to the DCs to efficiently travel to local lymph nodes and successfully present antigen to T cells, which generates effective cell-mediated immunity [4,5]. Dissemination of mycobacteria to the lymph node, which occurs in part via infected DCs, is an important precursor to T cell activation [6,7]. A deficiency of DCs, monocytes, B and NK cells (DCML deficiency), with an as yet unknown genetic basis, has recently been defined in four subjects. Two of these subjects succumbed to mycobacterial infection: one developed disseminated BCG-osis and the other was diagnosed with spontaneous *Mycobacterium kansasii *infection [8]. Similarly, mutations in interferon regulatory factor 8 (IRF8), described recently in three subjects, are associated with dendritic cell deficiency resulting in susceptibility to disseminated BCG-osis [9]

We and others have shown how macrophage cell death follows infection with Mtb [10-13]. This macrophage response has consequences for aspects of innate and cell-mediated immunity [14,15]. The impact of Mtb infection on DC survival, however, is poorly understood. Given the non-redundant role of DCs in mycobacterial immunity [9], and their identification as a target for novel therapies and vaccines [4,16-19], we sought to define the requirements and mechanism of DC cell death after infection with Mtb. By modelling human monocyte-derived DCs *in vitro*, we infected DCs with Mtb to assess phagocyte survival, and attendant caspase activity, cytokine production and mycobactericidal effect.

Our results show that Mtb infection drives DC maturation and death. As we found in macrophages [10], the cell death that follows Mtb H37Ra infection is caspase-independent and is not characterised by nuclear fragmentation. In fact, infected DC death proceeds without the activation of caspases. Increased cytokine production followed DC infection with Mtb, but isolated DCs were not able to kill intracellular bacilli. Such data is of value in projecting how manipulation of DCs for new therapeutic strategies can be modelled.

## Results

### Live *M. tuberculosis *infection causes dendritic cell death

**Figure 1:**
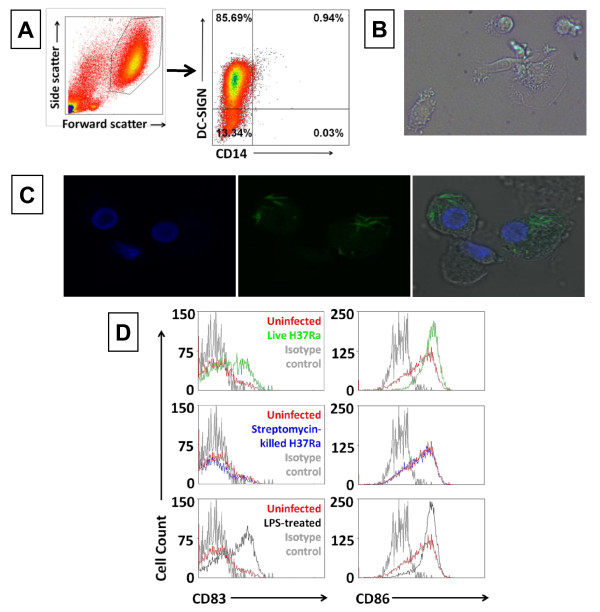
**Dendritic cells mature after they phagocytose *M. tuberculosis***. **A**. Human monocytes were separated from buffy coats by plastic adherence and cultured for 6 days in the presence of recombinant human IL-4 (40 ng/ml) and GM-CSF (50 ng/ml) to allow differentiation to DCs. Cells were analysed for CD14 and DC-SIGN expression by flow cytometry. DCs were CD14^- ^and DC-SIGN^+ ^(typically > 85% of gated cells; both before and after infection with Mtb). Plots show uninfected, immature DCs after 6 days of cytokine treatment from 1 representative donor of 3.. **B**. DCs were infected with live H37Ra at MOI 1 for 24 h and visualised by light microscopy. **C**. DCs were infected with live Mtb H37Rv at MOI 10 overnight. Bacteria were stained with auramine and nuclei with Hoechst and were visualised by confocal microscopy. Similar results were obtained with iH37Rv, live H37Ra and streptomycin-killed H37Ra (data not shown). **D**. DCs were infected with live Mtb H37Ra or streptomycin-killed H37Ra at MOI 1 for 48 h. Surface expression of CD83 and CD86 was assessed by flow cytometry. The histograms show 1 representative donor of 3.

Dendritic cells form an important link between the innate and the adaptive immune response, so their viability during infection may have consequences for the host. We prepared DCs from human blood as described in Methods. After 6 days' incubation, we reliably generated a population of DC-SIGN^+ ^CD14^- ^cells (Figure 1A) that also had a characteristic DC appearance under microscopy, displaying dendrites after exposure to Mtb H37Ra (Figure 1B) and H37Rv (data not shown). Great care was taken to confirm a reproducible MOI for live H37Ra and H37Rv, as well as dead Mtb bacilli, for each experiment, as discussed in Methods. Confocal microscopy (to assess phagocytosis of mycobacteria) and propidium iodide (PI) staining (to measure cell death) were carried out in DCs infected with either H37Ra or H37Rv. All other experiments were performed with H37Ra only. Figure 1C shows DCs infected with live H37Rv and stained with auramine to detect mycobacteria, and demonstrates that the mycobacteria were phagocytosed by the DCs. Similar results were obtained with γ-irradiated H37Rv, live H37Ra and streptomycin-killed H37Ra (data not shown).

Maturation was assessed in DCs infected with H37Ra. In controlled experiments, DCs were infected with live or dead Mtb H37Ra or at MOI 1for 24 h. Approximately 60% of cells had phagocytosed mycobacteria at this time point. The cells were washed to remove extracellular mycobacteria and either analysed or incubated for a further 24 or 48 h before analysis. DCs infected with live H37Ra displayed a mature phenotype, up-regulating CD83 and CD86 after 48 h infection with Mtb (Figure 1D). Streptomycin-killed H37Ra did not induce DC maturation.

**Figure 2:**
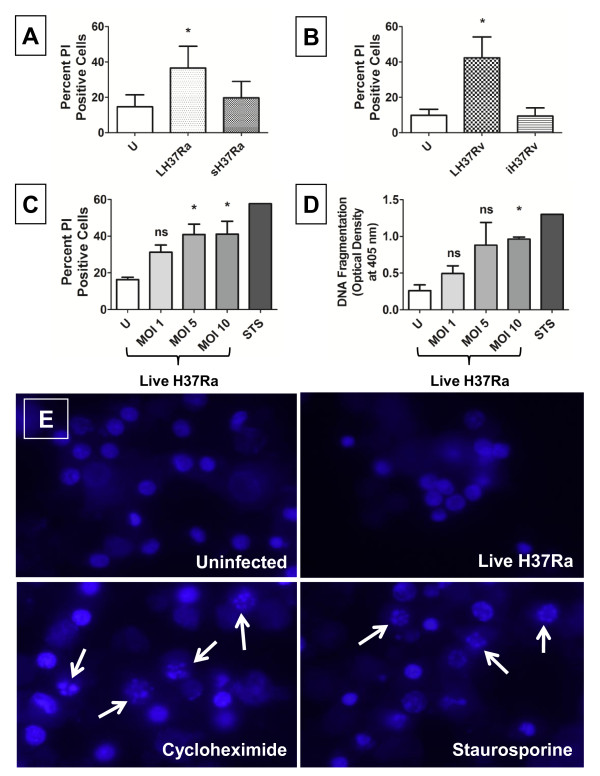
**Live *M. tuberculosis *infection causes dendritic cell death and DNA fragmentation, without nuclear fragmentation**. **A - B**. Dendritic cells (DCs) were infected, at MOI 10 with live/dead H37Ra or live/dead H37Rv. (U = uninfected, LH37Ra = live H37Ra, sH37Ra = streptomycin-killed H37Ra, LH37Rv = live H37Rv, iH37Rv = γ-irradiated H37Rv.) Cell death was measured by propidium iodide exclusion (A) 72 h post-infection or (B) 24 h post-infection on a GE IN Cell Analyzer 1000. (A - B) are means (± SEM) of 3 pooled donors. * *p *< 0.05 vs. Uninfected. **C**. DCs were infected with live H37Ra at MOI 1, 5 or 10. Cell death was measured by propidium iodide exclusion 72 h after infection. Staurosporine was used as a positive control for cell death. * *p *< 0.05 vs. uninfected, ns - not significantly different from uninfected. **D**. DCs were infected with live H37Ra at MOI 1, 5 or 10. DNA fragmentation was measured by Cell Death ELISA 72 h after infection. * *p *< 0.05 vs. Uninfected, ns - not significantly different from uninfected. **E**. DCs were infected with live H37Ra at MOI 10 for 72 h. Nuclei were stained with Hoechst and visualised by fluorescence microscopy. Cycloheximide and staurosporine were used as positive controls for nuclear fragmentation. (C - E) are 1 representative donor of 3, showing means (± SEM) of 3 independent wells.

To assess the relationship between intracellular infection and DC viability, we infected human monocyte-derived DCs with Mtb strains H37Ra and H37Rv. Viability of infected DCs (infected with 10 bacilli per cell) was assessed by PI exclusion and quantified on a GE IN Cell Analyzer 1000. Infection of DCs with either live strain was followed by cell death after 24-72 hours (Figures 2A and 2B), whereas dead bacilli (streptomycin-killed or irradiated) did not elicit this response. Incubation times with each strain were optimised to provide a significant increase in the percentage of PI positive cells above background (40-60%) while at the same time minimizing the cellular disintegration that occurs in the late stages of cell death and would lead to an underestimate of the numbers of dead cells. Longer incubation times led to the death of the majority of infected cells (> 95%). The virulent H37Rv strain induced cell death at a faster rate than an equivalent MOI of the attenuated H37Ra strain and as a consequence, the PI exclusion assay was carried out 24 h after infection in H37Rv-infected DCs and 72 h in H37Ra-infected cells. Cell death also occurred with live H37Ra infection at the lower MOIs of 1 and 5 after 72 h (Figure 2C).

Having established that reduced DC viability was dependent on infection with live mycobacteria, we then investigated the mechanism of cell death in H37Ra-infected DCs. We previously noted that macrophage cell death after Mtb infection results in DNA fragmentation. By ELISA, we could show that DNA fragmentation was also a feature of the DC response to viable Mtb H37Ra infection peaking at an MOI of 5 (Figure 2D).

Apoptosis results in nuclear condensation, pyknosis and, eventually, fragmentation of the nucleus into apoptotic bodies [20,21]. To determine whether this occurred during Mtb H37Ra infection, the nuclear morphology of DCs stained with Hoechst was examined by epifluorescent microscopy. The nuclei of infected cells did not undergo pyknosis or fragmentation and were similar in appearance to those of uninfected cells at 72 h after infection, a time at which they had undergone significant cell death. DCs treated with cycloheximide and staurosporine displayed extensive nuclear fragmentation, indicating that the cells are capable of undergoing this process when treated with apoptotic stimuli (Figure 2E).

### Dendritic cell death after *M. tuberculosis *H37Ra infection is caspase-independent and proceeds without the activation of caspase 3 and 7

**Figure 3:**
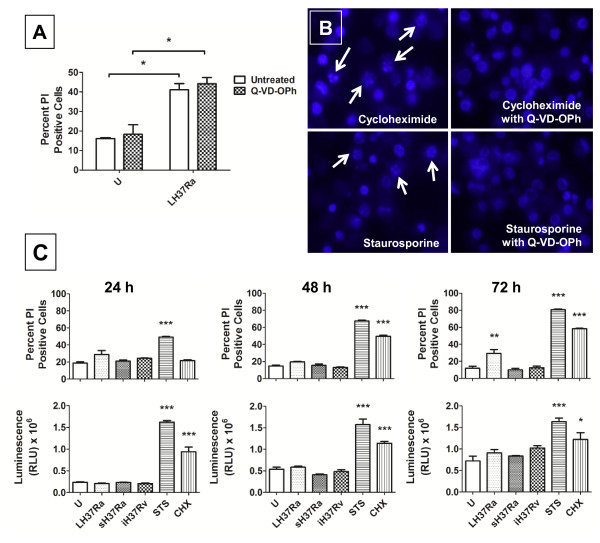
**Dendritic cell death after *M. tuberculosis *H37Ra infection is caspase-independent and proceeds without the activation of caspase 3 and 7**. **A**. DCs were infected with live Mtb H37Ra at MOI 10, in the presence or absence of the pan-caspase inhibitor, Q-VD-OPh (20 μM). Cell death was measured by propidium iodide exclusion 72 h post-infection. ** *p *< 0.01 vs. uninfected. Data represents means (± SEM) of 3 separate donors. **B**. As a positive control, DCs were treated with cycloheximide (5 μg/ml) or staurosporine (1 μM) in the presence or absence of Q-VD-OPh for 72 h. Nuclei were stained with Hoechst and visualised by fluorescence microscopy. **C**. DCs were infected with live/dead Mtb H37Ra or γ-irradiated H37Rv at MOI 10. Caspase 3/7 activity was assessed at 24 h, 48 h and 72 h in triplicate wells. Cell death was measured in parallel by propidium iodide exclusion(upper panels). (U = uninfected, LH37Ra = live H37Ra, sH37Ra = streptomycin-killed H37Ra, iH37Rv = γ-irradiated H37Rv, STS = staurosporine, CHX = cycloheximide.) * *p *< 0.05 vs. uninfected. ** *p *< 0.01 vs. uninfected. *** *p *< 0.001 vs. uninfected. Data represent means (± SEM) of 1 donor representative of 5.

Activation of caspases is considered to be essential for classical apoptosis [22]. Therefore, we sought to establish if DC death following Mtb infection was caspase dependent. Cells were treated with the pan-caspase inhibitor Q-VD-OPh and infected with H37Ra, at an MOI of 10, and cell death was assessed using IN Cell fluorescent microscopy and analysed as before. DCs were incubated with Q-VD-OPh 4 h prior to infection with Mtb and the inhibitor was replenished every 24 h during the experiment. Cell death was assessed at 72 h post-infection with H37Ra. As can be seen, the inhibition of caspases by Q-VD-OPh did not interfere with the level of cell death after Mtb infection (Figure 3A), although the inhibitor did prevent the apoptosis induced by cycloheximide and staurosporine (Figure 3B) [23].

Our results so far indicated that H37Ra-infected DC death occurred with DNA fragmentation, but without nuclear karyorrhexis and was caspase-independent. Caspase-independent cell death can occur with or without caspase activation, depending on the mechanism of cell death [24]. In order to more closely examine the role of caspases in DC death induced by Mtb H37Ra infection, we analysed the activity of the executioner caspases 3/7 in parallel with cell death at 24 h, 48 h and 72 h post-infection with Mtb (Figure 3C). Staurosporine (24 h treatment at all time points) and cycloheximide (24, 48 and 72 h treatment in parallel with infection) were used as positive controls for caspase activity, inducing increased caspase 3/7 activity at all time points examined (Figure 3C). Caspase activity was measured before and after significant cell death had occurred. Cell death due to Mtb H37Ra was apparent at 72 h post-infection (Figure 3C) and occurred with live Mtb infection only, as in our previous experiments (Figure 2). Caspases 3/7 were not active above levels recorded in uninfected DCs at any time point examined, indicating that these caspases are not activated during DC death after Mtb H37Ra infection.

### Secretion of cytokines by Mtb H37Ra-infected dendritic cells

**Figure 4:**
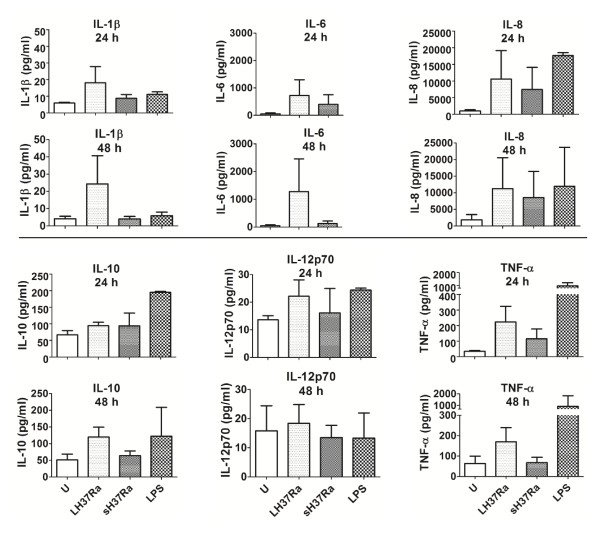
**Dying *M. tuberculosis*-infected DCs secrete cytokines**. DCs were infected with live/dead Mtb H37Ra at MOI 1 for 24 h or 48 h, or treated with LPS (1 μg/ml) for 24 h. (U = uninfected, LH37Ra = live H37Ra, sH37Ra = streptomycin-killed H37Ra.) Cytokine levels were measured in cell-free supernatants by ELISA. Data were analysed using the Friedman test followed by Dunn's Multiple Comparison test and represent the means (± SEM) of 3 individual donors.

Although macrophages and neutrophils die after Mtb infection, these dying and dead cells have been shown to play a role in host immune responses [11,25-29]. To test if infected DCs contribute to immunity by secreting cytokines, we infected DCs with H37Ra at a low MOI of 1 bacillus per DC and assessed cytokine production (assayed by Meso Scale Discovery multiplex ELISA). Low MOI caused 40-60% death after 72 h (shown in Figure 2C) and was chosen to allow assessment of cytokine release at 24 and 48 hours post-infection before excessive cell death had occurred. Infection of DCs from each donor (n = 3) with H37Ra consistently stimulated the release of pro- and anti-inflammatory cytokines (Figure 4) including TNFα, IL-6, IL-8, IL-10, IL-1β and a modest increase in secretion of IL-12p70. There was a tendency for DCs infected with killed H37Ra to produce less IL-10, TNFα, IL-6 and IL-1β than cells infected with live H37Ra but these results did not reach statistical significance when the data was pooled due to donor variation. Other cytokines were unchanged after infection (IL-2, IFN-γ, IL-5 and IL-13; data not shown).

### Dendritic cells are permissive for growth of *Mycobacterium tuberculosis *H37Ra

**Figure 5:**
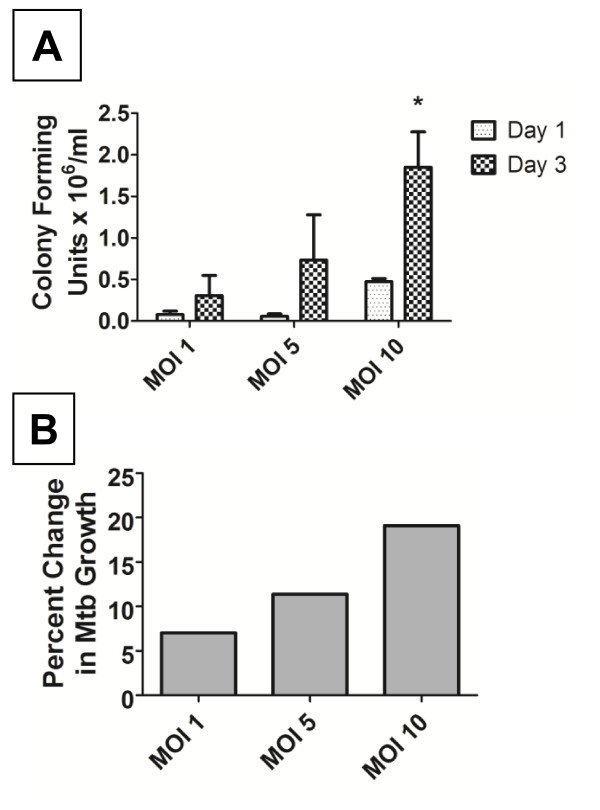
**Dendritic cells are permissive for growth of *M. tuberculosis *H37Ra**. **A**. DCs were infected with live Mtb H37Ra for 24 h (Day 1) or 72 h (Day 3), at varying MOI. Colony-forming units were counted after 21 days. The graph represents the mean (± SEM) of 3 donors. * *p *< 0.05 vs. Day 1. **B**. DCs were infected as above for 3 days and incubated in a bioMérieux BacT/ALERT 3D automated microbial detection system. Detection of bacterial growth was labelled 'positive' and time to reach positivity (TTP) was recorded. Percentage time to positivity was calculated using the formula: ((TTPDay1-TTPDay3)/TTPDay1) × 100. A positive change in percentage time to positivity was indicative of bacterial growth. The results shown are from 1 representative donor of 3.

Alveolar macrophages also die after Mtb infection and yet are capable of restricting the growth of Mtb [30]. Dendritic cells are the professional cell required to activate CD4^+ ^and CD8^+ ^T cells to enable killing of intracellular Mtb, yet infected DCs could also limit Mtb growth. There are conflicting reports within the literature regarding the fate of Mtb strains within DCs. In the present study the ability of Mtb H37Ra to replicate within human DCs, in the presence of GM-CSF and IL-4, was studied using two separate methods: colony forming unit (CFU) counts and the bioMérieux BacT/ALERT 3D automated microbial detection system (Figure 5). At MOIs of 1, 5 and 10 bacilli per DC, we confirmed that Mtb grew over 3 days using CFU analysis on Middlebrook agar. At the same time point, we saw a similar dose-response for bacillary growth using liquid Middlebrook media in a BacT/ALERT system; a growth index was generated using 'time to positivity' data (see Methods). Apoptosis has been linked to improved mycobactericidal effects in macrophages [11,31,32]; whereas we found that Mtb replicates within DCs despite (or perhaps because of) the abundant non-apoptotic cell death that occurs during infection.

## Discussion

We investigated the impact of Mtb infection on the viability of human monocyte-derived dendritic cells. We found that DC death followed infection with both the H37Ra and H37Rv strains of Mtb, required viable bacilli, and could be detected at 24 hours co-incubation. The type of cell death was atypical of apoptosis, because it lacked nuclear fragmentation. Cell death due to infection with H37Ra was caspase-independent, although it did proceed with DNA fragmentation. Caspase activation was not detected by substrate assay analysis. Although this type of cell death did not interfere with earlier DC maturation events or cytokine release, it was not associated with any detectable mycobactericidal effect of DCs.

With regard to mycobactericidal effect, DC death differs from H37Ra-infected macrophage cell death, which can kill the invading parasite [30]. In murine DCs the consequences of cell death after infection with *Legionella pneumophila *link caspase activity and bacterial killing [33], however we did not see caspase 3 or 7 activity, or association with Mtb killing. Other groups have examined DC mycobactericidal capacity using different models, with differing results [34-36]. Fortsch et al. and Bodnar et al. [34,35] found that DCs were permissive for growth of intracellular Mtb, while Tailleux et al. [36] reported constraint of Mtb replication within DCs without the addition of IFN-γ. The proposed difference in findings was suggested to be due to removal of the cytokines GM-CSF and IL-4 from DCs upon infection with Mtb. We maintained the GM-CSF/IL-4 supplementation of our DCs in culture to maintain the DC phenotype, and these factors did not support infected DC viability or ability to limit intracellular bacterial replication. Similar findings were reported in murine Mtb-infected DCs maintained in IL-4, which were unable to control mycobacterial growth in the absence of exogenous IFN-γ [35]. Our experiment models the early stages of Mtb infection in the lung where newly arrived DCs may become infected before being activated by exposure to T_H_1 cytokines allowing uncontrolled proliferation of mycobacteria. After the initiation of a T cell response and the formation of the granuloma infected DCs are more likely to be exposed to IFNγ and may be better able to control the growth of mycobacteria.

It is perhaps not surprising that DCs failed to kill bacilli by themselves, without T cell help. Other consequences of macrophage cell death after Mtb infection have been described, such as better antigen presentation [14,37]. DCs play a key role in antigen presentation, which results in activation of T cell populations that can lead to efficient phagocyte killing of the intracellular bacillus, via granulysin-induced phagocyte death, or by cytokine release (e.g. IFN-γ) that supports the mycobactericidal capacity of phagocytes [38-41]. Although outside the scope of this current article, it is possible that dying DCs share some properties of dying macrophages, and contribute to this T cell response.

In the present study we found that both the attenuated H37Ra and virulent H37Rv strains cause death of human DCs. The caspase-independent cell death we report in H37Ra-infected DCs appears to be neither apoptosis nor pyroptosis (both of which require caspase activity) [22,42]. There are various modes of non-apoptotic cell death, such as pyronecrosis and necroptosis, which can occur without caspase activation. The way in which cells die shapes the response of the immune system; death can be immunogenic, tolerogenic or silent [43,44]. Therefore, the type of cell death undergone by Mtb-infected DCs is of interest, as it may either support or inhibit cytotoxic and helper T cell responses. Macrophage apoptosis appears to be beneficial for the host response to tuberculosis by having direct bactericidal effects on intracellular mycobacteria and also in the stimulation of protective immunity. The genome of *M. tuberculosis *contains genes that actively inhibit macrophage apoptosis and enhance its intracellular survival, including *nuoG*, *pknE *and *secA2 *[45]. It is likely that the products of these genes would also inhibit apoptosis of DCs, possibly steering the cells towards the non-apoptotic mode of cell death seen in the present study. Interestingly, foamy macrophages (which are positive for DC markers) in granulomas in the lungs of mice infected with *M. tuberculosis *have been found to express high levels of TNFR-associated factors (TRAFs) 1-3 which are associated with resistance to apoptosis [46]. Although H37Ra and H37Rv are highly related, being derived from the same parental H37 strain, they differ in important respects at the genetic [47], transcriptional [48] and post transcriptional [49] levels. As a result H37Ra displays several characteristics that are different from H37Rv (e.g. variations in PE/PPE/PE-PGRS proteins [47], decreased survival inside human macrophages [50,51], differences in the composition of mannose caps on lipoarabinomannin [52] and impaired ability to secrete ESAT 6 [49]) each of which could have an impact on the mode of cell death [53,54]. Indeed, similar to our previous finding in human macrophages [10], H37Rv infection killed DCs at a significantly faster rate than H37Ra. Further work will be needed to determine whether infection of DCs with H37Rv causes a similar caspase-independent mode of cell death.

Caspases can have variable effects on the immunogenic potential of dying cells. Exposure or release of damage-associated molecular pattern molecules (DAMPs), such as calreticulin, high-mobility group box 1 (HMGB1) or heat-shock proteins, by dying cells is associated with immunogenic cell death. Calreticulin exposure has been shown to be of particular importance in the induction of immunogenic cell death [55]. Exposure of calreticulin is caspase-dependent; however caspases can also mitigate the pro-inflammatory release of DAMPs from dying cells and cell death that proceeds without the activity of caspases may generate more immune-activating DAMPs [43,56]. Such an outcome might benefit the host response. These DAMPs could escape from the cell, unimpeded by caspase-neutralisation, and proceed to work in concert with the pro-inflammatory cytokine profile we observed, to generate a better inflammatory response in the lymph node. Yet, cross-priming of T cells is improved by caspase-dependent macrophage apoptosis [14,57]. Whether DC death that occurs without caspase activation can elicit a CD8^+ ^T cell response remains to be seen.

It is also possible that DC death could interfere with important DC functions such as migration to local lymph nodes for efficient antigen presentation. Others have shown that DC migration to local lymph nodes is impaired in Mtb infection [58,59], which would delay stimulation of T cell responses. Although DC death could contribute to this phenotype, DC migration to the draining lymph node can take 18 hours *in vivo *after challenge with Mtb [60]. Although we cannot extrapolate directly from our *in vitro *experiments to the complex environment that these cell are exposed to *in vivo*, infected DCs are known to traffic from the lung to lymph nodes [58]. At low MOI, the DC may arrive at the node before undergoing death in an environment where cell death can contribute to antigen cross-presentation. Elimination of the infected DCs could also deprive the host response of an important source of cytokines and antigen presentation; though data from Alaniz et al. suggest that DCs can serve, like macrophages, as a niche cell that promotes intracellular bacterial replication [61]. Mtb-infected DCs produced IL-1β, IL-6, IL-8, IL-10, IL-12p70 and TNF-α as reported previously [62-66] despite the fact that the majority of the cells eventually die. The cytokine profile of Mtb-infected DCs would successfully drive differentiation of T_H_1 and T_H_17 responses [67].

Mtb and the human immune system have co-evolved, so that one third of the global population has been colonised by this pathogen, yet the immune system is adequate at preventing disease 90% of the time [1,2]. The central cell that regulates this host response is the dendritic cell, and consequently it is increasingly viewed as a target for new therapeutic and vaccine strategies [19,68]. It is hoped that our description of the DC death response to Mtb infection - as pro-inflammatory, and without the activation of caspases - will inform further research that defines the T cell consequences of this innate response. An improved understanding of these immune processes should help advance translational research to address this global killer.

## Conclusions

We have presented evidence that DCs undergo cell death after infection with Mtb *in vitro*, just as macrophages do. In H37Ra infection this non-apoptotic response does not limit the viability of the infecting bacillus, yet it does not interfere with DC maturation or cytokine production, as previously reported. The lack of caspase activity seen may also contribute to the host response by allowing DAMPS to drive anti-TB immunity, without neutralisation by these important proteases. Further work is needed to determine whether the virulent strain H37Rv induces a similar non-apoptotic form of cell death in human DCs.

## Methods

### Mycobacteria

*M. tuberculosis *strains H37Ra and H37Rv were obtained from the American Type Culture Collection (Manassas, VA). Mycobacteria were propagated in Middlebrook 7H9 broth (Difco/Becton Dickinson, Sparks, MD) supplemented with albumin-dextrose-catalase supplement (Becton Dickinson) and 0.05% Tween 80 (Difco). Aliquots were stored at -80°C, thawed and grown to log phase in Middlebrook 7H9 medium before use.

### Inactivation of mycobacteria with streptomycin

Log-phase H37Ra were treated with streptomycin sulphate (Sigma, St. Louis, MO; 0.1 mg/ml) for 48 h prior to infection. Streptomycin was thoroughly washed from mycobacteria prior to DC infection.

### Gamma-irradiated H37Rv

Obtained through the NIH Biodefense and Emerging Infections Research Resources Repository, NIAID, NIH: *Mycobacterium tuberculosis*, Strain H37Rv, Gamma-Irradiated Whole Cells, NR-14819.

### Cell Culture

Peripheral blood mononuclear cells (PBMCs) were isolated from buffy coats of anonymous healthy donors (provided, with permission, from the Irish Blood Transfusion Service). The PPD status of donors was unknown. PBMCs were separated by density centrifugation on Lymphoprep (Axis-Shield, Oslo, Norway), washed and re-suspended in serum-free RPMI 1640 (Gibco, Invitrogen, Carlsbad, CA; for plastic adherence monocyte separation) or in PBS (Sigma) with 2% defined foetal bovine serum (FBS; HyClone, Thermo Fisher Scientific, Waltham, MA) and 1 mM EDTA (Sigma) (for immunomagnetic negative selection). Monocytes were isolated by plastic adherence, or by negative selection using the immunomagnetic negative selection EasySep Human Monocyte Enrichment Kit (STEMCELL Technologies, Vancouver, BC), as per manufacturer's instructions. For plastic adherence separation, PBMCs were incubated at 37°C for 2 h in serum-free RPMI. After incubation, unwanted cells were thoroughly washed from the adherent monocytes, which were then incubated in DC medium: RPMI supplemented with 10% defined FBS, 40 ng/ml recombinant human IL-4 and 50 ng/ml recombinant human GM-CSF (both ImmunoTools, Friesoythe, Germany). For immunomagnetic negative selection, PBMCs were incubated with magnetic particles coated with antibodies targeting unwanted cells (anti- CD2, CD3, CD16, CD19, CD20, CD56, CD66b, CD123 and glycophorin A). Labelled cells were magnetically separated and discarded, isolating the unlabelled monocytes. Monocytes were then incubated in DC medium. DCs were seeded on 24-, 48- or 96-well culture dishes at a density of 1 × 10^6 ^cells/ml and cultured for 6 days prior to infection with *M. tuberculosis*. The medium, containing fresh cytokines, was replaced every 2 to 3 days. Cytokines were also replenished 24 h after infection with *M. tuberculosis*, to maintain cytokine activity and DC phenotype throughout Mtb infection.

### *In vitro *infection of DCs with *M. tuberculosis*

On the day of infection, mycobacteria were centrifuged at 3,800 rpm for 10 min and re-suspended in RPMI 1640 containing 10% defined FBS. Clumps were dispersed by passing the bacterial suspension through a 25 gauge needle eight times, and the sample was centrifuged at 800 rpm for 3 min to remove any remaining clumps. To determine the amount of Mtb necessary to achieve the required MOI, a CrystalSpec nephelometer (BD Diagnostic Systems, Sparks, MD) was used to estimate bacterial numbers in *M. tuberculosis *suspension. (Nephelometer bacterial number estimates was validated by counting colony-forming units (CFU) of bacterial suspension, plated on Middlebrook 7H10 agar plates, after 14 days). MOI were then calculated as bacteria per cell. DCs were infected at various MOI for 24 h, and extracellular bacteria were then removed by twice exchanging the medium with fresh DC medium. After 24 h infection, slides were prepared for acid-fast bacteria (AFB) staining to confirm phagocytosis. The cells were fixed for 10 min (H37Ra) or 24 h (H37Rv) in 2% paraformaldehyde (Sigma), applied to glass slides and left to air dry overnight. Slides were then stained with modified auramine O stain (Scientific Device Laboratory, Des Plaines, IL) for acid-fast bacteria. DC nuclei were counterstained with 10 μg of Hoechst 33358/ml (Sigma). The number of bacilli per cell was determined by observing the slides under an inverted fluorescence microscope (Olympus IX51, Olympus Corporation, Center Valley, PA). After infection, DCs were maintained in culture at 37°C for 1 to 3 days before harvesting.

### Propidium iodide staining for IN Cell Analyzer viability assessment

Viability was assessed using the propidium iodide (PI) exclusion method for plasma membrane integrity of cells, and the nuclei were counterstained with Hoechst. Cells were incubated with 10 μg of PI/ml, Hoechst 33342 (10 μg/ml), and Hoechst 33358 (10 μg/ml) for 30 min at room temperature. The number of PI-positive cells relative to the total number of nuclei per field was counted by automated fluorescence microscopy using the IN Cell Analyzer 1000 and IN Cell Investigator software (GE Healthcare, Pittsburgh, PA). Each condition was assayed in triplicate, and 8 fields were counted in each well. Staurosporine (Sigma) (1 μM, diluted in serum-free RPMI) was applied for 24 h as a positive control for cell death.

### DNA fragmentation (cell death ELISA)

The cell death detection ELISA^PLUS ^kit (Roche Applied Science, Mannheim, Germany) was used to quantify *M. tuberculosis*-induced DNA fragmentation, as recommended by the manufacturer. Briefly, 1-3 days after infection, 48-well plates were centrifuged at 200 × *g *to sediment detached cells, the medium was discarded, and the cells were lysed. The lysate was subjected to antigen capture enzyme-linked immunosorbent assay (ELISA) to measure free nucleosomes, and the optical density at 405 nm (OD405) was read on a Victor^2 ^plate reader (Wallac/Perkin Elmer, Waltham, MA). Triplicate wells were assayed for each condition. Staurosporine (Sigma) (1 μM, diluted in serum-free RPMI) was applied for 24 h as a positive control for DNA fragmentation.

### Caspase Inhibition

The pan-caspase inhibitor, Q-VD-OPh (20 μM; Enzo Life Sciences AG, Lausen, Switzerland), was applied to DCs 4 h prior to infection with H37Ra and replenished every 24 h throughout the duration of infection

### Caspase-Glo Assay

Caspase 3/7 activity was measured using the luminescent Caspase-Glo assay system (Promega, Madison, WI). DCs were cultured in 96-well plates and the assays were carried out in a total volume of 200 μl. After equilibration to room temperature, Caspase-Glo reagent was added to each well and gently mixed using a plate shaker at 300 rpm for 30 s. The plate was incubated at room temperature for 30 minutes and luminescence was then measured using a Victor^2 ^plate reader.

### Laser Scanning Confocal Microscopy

Following infection, DCs were fixed for 10 min (H37Ra) or 24 h (H37Rv) in 2% paraformaldehyde (Sigma), applied to glass slides and left to air dry overnight. The cells were then stained with modified auramine O stain for acid-fast bacteria and DC nuclei were counterstained with 10 μg/ml of Hoechst 33358. The slides were analysed using a Zeiss LSM 510 laser confocal microscope equipped with an Argon (488 nm excitation line; 510 nm emission detection) laser and a diode pulsed solid state laser (excitation 561 nm; emission 572 nm long pass filter) (Carl Zeiss MicroImaging GmbH, Oberkochen, Germany). Images were generated and viewed using LSM Image Browser (Carl Zeiss MicroImaging).

### Flow Cytometry

Dendritic cell surface markers were analysed by flow cytometry on a CyAn ADP flow cytometer (Dako/Beckman Coulter). Dendritic cells were infected with live H37Ra, or streptomycin-killed H37Ra at MOI 1 for 24 or 48 h. As a positive control for maturation, uninfected DCs were treated with LPS (Sigma; 1 μg/ml) for 24 h prior to staining for flow cytometry. Cells were incubated with antibodies for 30 min and fixed with 2% paraformaldehyde for at least 1 h prior to flow cytometry.

The following antibodies were used: FITC mouse anti-human CD14, FITC mouse IgG2a, κ isotype control, PE mouse anti-human CD209 (DC-SIGN), PE mouse IgG2b κ isotype control, APC mouse anti-human CD83, APC mouse IgG1 κ isotype control, FITC mouse anti-human CD86, FITC mouse IgG1 κ isotype control (all BD Pharmingen, San Diego, CA).

Cells were gated on forward scatter and side scatter to exclude clumps and debris. DCs were CD14^- ^and DC-SIGN^+ ^(constituting approximately 90% of gated cells). Results were analysed using Summit software version 4.3 (Dako/Beckman Coulter).

### Cytokine Analysis

Dendritic cells were infected with live H37Ra or streptomycin-killed H37Ra at MOI 1 for 24 or 48 h. LPS was applied for 24 h (Sigma; 1 μg/ml) as a positive control for DC maturation and cytokine secretion. Cytokine secretion was measured in cell-free supernatants by ELISA using the Meso Scale Discovery SECTOR Imager 2400 and the following assays: human IL-6 assay, and human Th1/Th2 10-cytokine multiplex assay, capable of detecting IFN-γ, IL-1β, IL-10, IL-12p70, IL-13, IL-2, IL-4, IL-5, IL-8 and TNF-α (Meso Scale Discovery, Gaithersburg, MD). IL-4 measurements were disregarded, as DCs were maintained in culture with exogenous IL-4, rendering it impossible to distinguish levels secreted by the cells themselves.

### Colony forming units and BacT/ALERT 3D

Dendritic cells were harvested 24 h or 72 h after infection with *M. tuberculosis*. Cells were centrifuged and washed 3 times at 800 rpm to remove extracellular bacteria. The cells were lysed in 0.1% Triton X-100 (Sigma) for 10 min. The resultant bacterial suspension was then passed through a 25 gauge needle eight times to disperse clumps. The bacilli were serially diluted x10^-1 ^- x10^-5 ^in Middlebrook 7H9 medium and plated on Middlebrook 7H10 agar (Difco) supplemented with oleic acid-albumin-dextrose-catalase (Becton Dickinson) and cycloheximide (Sigma), or inoculated into BacT/ALERT MP bottles (bioMérieux, Durham, NC). Agar plates were incubated at 37°C for 14 - 21 days and colony forming units were counted. BacT/ALERT MP bottles were incubated in a BacT/ALERT 3D automated microbial detection system (bioMérieux) and time to reach positivity was recorded, and a growth index was calculated, using the equation ((TTPDay1-TTPDay3)/TTPDay1)x100 as we have already published for the BD BACTEC liquid culture platform [69]. In this equation TTP Day 1 is the time to culture positivity for infected DC lysates at Day 1, and TTP Day 3 is the time to positivity for infected DC lysates at Day 3.

### Statistical analysis

Results are expressed as means ± the standard errors of the mean (SEM). The data were analyzed with GraphPad Prism 5 software (GraphPad Software, Inc., La Jolla, CA) statistical software using by repeated measures ANOVA with Tukey's post test, or (where stated) by the Friedman test followed by Dunns multiple comparison test. A *P *value of < 0.05 was considered statistically significant. Graphs were compiled using GraphPad Prism 5 software.

## Authors' contributions

RCMR performed the experiments and prepared the figures; MPOS performed the cytokine ELISAs; RCMR and MPOS analysed the data; MPOS and JK conceived of and designed the study; RCMR, MPOS and JK wrote the manuscript. All authors read and approved the final manuscript.
